# Targeting the BCL2 Family: Advances and Challenges in BH3 Mimetic-Based Therapies

**DOI:** 10.3390/ijms26209859

**Published:** 2025-10-10

**Authors:** Nabanita Mukherjee, James Sheetz, Yiqun G. Shellman

**Affiliations:** 1 Department of Dermatology, University of Colorado Anschutz Medical Campus, Aurora, CO 80045, USA; 2 Sidney Kimmel Medical College, Thomas Jefferson University, Philadelphia, PA 19107, USA; 3 Gates Institute, University of Colorado Anschutz Medical Campus, Aurora, CO 80045, USA; 4 Rocky Mountain Regional VA Medical Center, Aurora, CO 80045, USA

**Keywords:** BCL-2, MCL-1, BCL-XL, apoptosis, resistance, immunomodulatory, non-apoptotic functions, anti-PD1, BFL-1, A1, immune checkpoint inhibitors, ICI

## Abstract

The BCL2 family of proteins plays a pivotal role in regulating apoptosis and cellular homeostasis, making them critical therapeutic targets in cancer and other diseases characterized by pathological cell survival. BH3 mimetics, small molecules that selectively inhibit anti-apoptotic BCL2 family members, have achieved significant clinical success, particularly in hematologic malignancies. However, several challenges remain, including resistance mechanisms, toxicity (such as MCL1 inhibitor-associated cardiotoxicity), and the intricate balance between apoptotic and non-apoptotic functions. This review provides a comprehensive overview of BCL2 family biology, the development and clinical application and outcomes of BH3 mimetics, and the emerging resistance mechanism known as double-bolt locking. We also examine strategies to overcome resistance, including combination therapies and immunomodulatory approaches. Beyond oncology, we highlight the expanding therapeutic potential of BH3 mimetics in autoimmune, fibrotic, and infectious diseases, as well as regenerative and anti-aging medicine. Finally, we discuss predictive biomarkers and tissue-specific responses that inform precision therapy. Together, these insights underscore the promise of BH3 mimetics and the need for continued multidisciplinary research to optimize their clinical impact.

## 1. Introduction

The BCL2 family of proteins plays a central role in regulating programmed cell death, or apoptosis, through interactions between its pro- and anti-apoptotic members. These proteins maintain a delicate balance of signals that determine whether a cell survives stress or damage. When this balance is disrupted, it can contribute to disease. For instance, overexpression of anti-apoptotic BCL2 proteins is linked to cancer development, resistance to therapy, and disease progression.

This critical role has made anti-apoptotic BCL2 family members major targets in the development of BH3 mimetics, a class of drugs designed to mimic natural pro-apoptotic proteins and selectively inhibit anti-apoptotic BCL2 proteins. Venetoclax, a leading BH3 mimetic, has shown promise in clinical settings. However, its use has also revealed significant challenges, particularly the rapid emergence of drug resistance.

This review provides a comprehensive overview of the fundamental roles and mechanisms of the BCL2 family and BH3 mimetics. It highlights recent advances in our understanding, including clinical outcomes, novel mechanisms, and emerging therapeutic applications. Notably, it discusses the double-bolt locking mechanism, which confers structural resistance to BH3 mimetics. In particular, this review highlights recent novel approaches, such as combining BH3 mimetics with immunomodulatory agents to enhance immunotherapy efficacy and overcome resistance. Additionally, it explores the potential applications of BH3 mimetics beyond oncology, including in autoimmune diseases, fibrosis, and sepsis. In these contexts, their immune-modulating and senolytic properties present therapeutic opportunities that have, until now, received limited attention. Finally, we explore the non-apoptotic functions of BCL2 family proteins and the ongoing search for predictive biomarkers to guide treatment decisions. Together, this review aims to provide a broad and insightful perspective on the therapeutic potential and current challenges of targeting the BCL2 family across diverse disease contexts.

## 2. Targeting the BCL2 Protein Family with BH3 Mimetics in Cancer Therapy

### 2.1. The BCL2 Protein Family and Its Role in Apoptosis

The BCL2 protein family is an evolutionarily conserved group that regulates the intrinsic (mitochondrial) apoptosis pathway. These proteins are found across species—from primitive metazoans such as sponges to mammals [1,2]. In humans, eighteen BCL2 family members have been identified, though not all are fully characterized.

Structurally, BCL2 family proteins share between one and four BCL2 homology (BH) domains (BH1–BH4), along with a C-terminal transmembrane domain that typically localizes them to the mitochondrial outer membrane. Among these, the BH3 domain is particularly important for mediating protein–protein interactions within the family and is essential for both pro- and anti-apoptotic functions [3,4,5,6,7,8].

**Figure 1:**
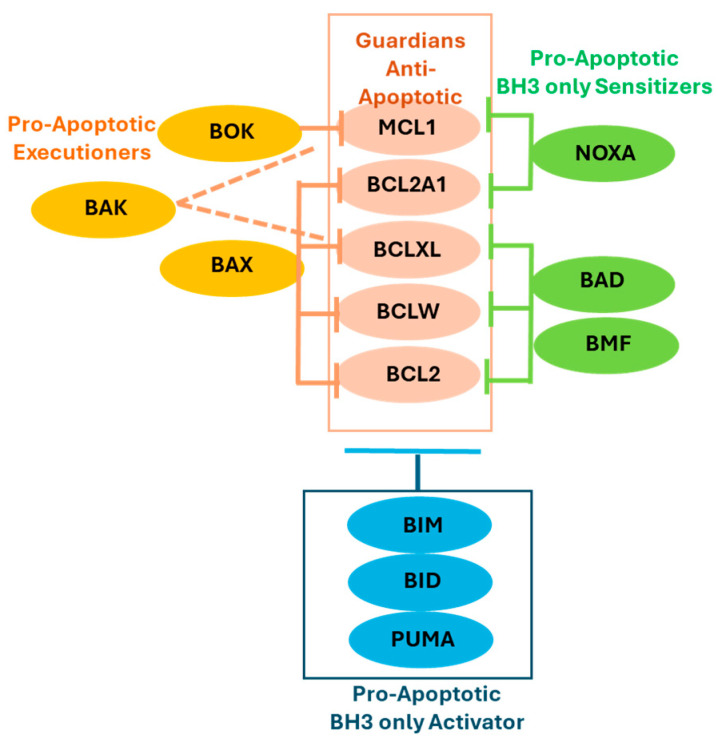
Interactions and roles of BCL2 family proteins in apoptosis: The major anti-apoptotic members of the BCL2 family function as guardians by binding and sequestering pro-apoptotic initiators and executioners through high-affinity interactions with their BH3 domains. The remaining pro-apoptotic members are categorized into three functional groups: activators, sensitizers, and executioners. Interactions among the members are selective rather than uniform.

Functionally, the BCL2 family is divided into two main groups: anti-apoptotic (pro-survival) proteins and pro-apoptotic proteins. The pro-apoptotic members are further classified as initiators (activators or sensitizers) and executioners. Figure 1 provides the details of the major pro- and anti-apoptotic proteins.

#### 2.1.1. Anti-Apoptotic Guardians

The major anti-apoptotic proteins, BCL2, BCLXL, MCL1, BCLW, and BCL2A1 (also known as BFL1), are often referred to as the “guardians” of the cell. These proteins inhibit apoptosis by binding and sequestering pro-apoptotic counterparts, particularly BH3-only initiators and the executioners. These anti-apoptotic proteins share a common structure that includes four BH domains and a key feature of their helical bundle structure, which forms a hydrophobic groove that binds the BH3 domain of pro-apoptotic proteins with high affinity. This interaction prevents mitochondrial outer membrane permeabilization (MOMP), halting the release of cytochrome c and blocking caspase activation. The BH4 domain in anti-apoptotic members also plays a critical role in stabilizing these interactions. Interestingly, loss of the BH4 domain can convert anti-apoptotic proteins into pro-apoptotic ones [9,10].

Importantly, interactions among BCL2 family members are not uniform; each anti-apoptotic protein exhibits selective binding preferences for specific pro-apoptotic partners. For example, BCL2 preferentially binds BIM, PUMA, BAD, and BAX. BCLXL binds BIM, BAD, BAX, and BAK. MCL1 binds NOXA, BIM, PUMA, and BAK, and NOXA binds almost exclusively to MCL1, targeting it for degradation. BCLW interacts with BAX and BAK, and several BH3-only proteins such as BAD, tBID, BIM, PUMA, BMF, and BIK [11,12,13,14]. BCL2A1 sequesters BIM, BID, and NOXA. This selectivity is determined by the compatibility between BH3 domains and the hydrophobic grooves of anti-apoptotic proteins. Overexpression of these guardians is common in cancer and contributes to therapy resistance [4,7,15].

BCLB (also known as BCL2L10) is a less characterized member of this group. It is expressed in various normal tissues and tumors and is generally considered anti-apoptotic. However, its function appears to be context-dependent and remains incompletely understood [2,16,17].

#### 2.1.2. Pro-Apoptotic Members

Pro-apoptotic initiators respond to cellular stress or damage and trigger apoptosis by interacting with other family members. These initiators fall into two subgroups: activators (e.g., BIM, PUMA, BID), which directly activate executioner proteins, and sensitizers (e.g., BAD, NOXA, BMF), which neutralize anti-apoptotic proteins by binding their BH3 domains but do not directly engage executioners. Executioner proteins BAX, BAK, and BOK carry out the final steps of apoptosis [4,7,18].

While all initiators can bind at least one anti-apoptotic protein, only a subset, such as BID, BIM, and PUMA, can directly activate executioners. Sensitizers lack this ability but play a critical role by displacing activators from anti-apoptotic proteins, thereby promoting apoptosis [4,6,19,20].

Emerging evidence suggests that the traditional division between activators and sensitizers is more fluid than previously thought. For example, NOXA, long considered a sensitizer, has been shown to bind BAK, indicating overlapping functions [21]. Initiators also vary in specificity, with some targeting a single anti-apoptotic protein and others binding multiple. These interactions are typically mediated by the BH3 domain, which adopts an α-helical structure to facilitate binding. BID is a notable exception, also utilizing its BH4 domain for this function [6,20,22].

Once anti-apoptotic proteins are neutralized, executioners (BAX, BAK, BOK) become activated and induce mitochondrial outer MOMP, leading to cytochrome c release, caspase activation, and cell death. These proteins represent the terminal effectors of the intrinsic apoptotic pathway [20,23].

Although best known for their roles in apoptosis, BCL2 family proteins also participate in non-apoptotic processes, including development, tissue homeostasis, autophagy, and immune regulation (see Section 6 below for more details). Table 1 summarizes the key BCL2 family members and their functions.

### 2.2. Roles of BCL2 Family Proteins in Cancer

**Figure 2:**
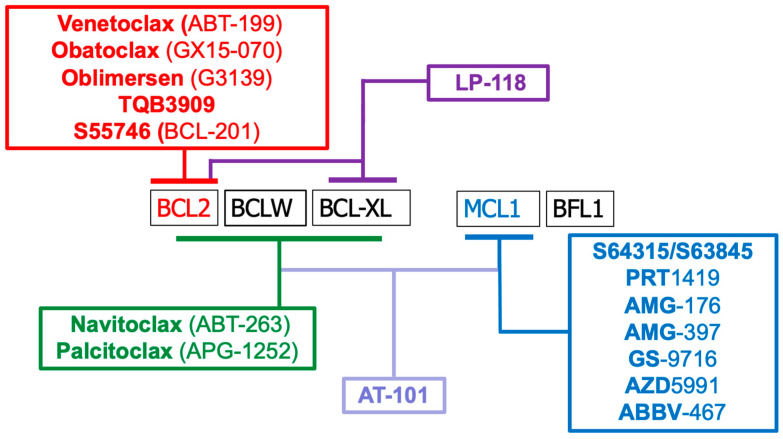
BH3 mimetics that have entered clinical use or trials, along with their target specificities. Some compounds exhibit overlapping activity against multiple BCL2 family proteins. Detailed descriptions of each drug are provided in Table 2.

Upregulation of anti-apoptotic members is a hallmark of many cancers, enabling malignant cells to evade apoptosis and develop resistance to therapy. BCL2 was first identified in follicular lymphoma due to a t(14;18) translocation that drives its constitutive expression [24]. Elevated BCL2 levels are also seen in chronic lymphocytic leukemia (CLL), diffuse large B-cell lymphoma (DLBCL), and acute myeloid leukemia (AML), contributing to poor treatment response [25]. MCL1, regulated by survival pathways like PI3K/AKT and ERK, is frequently amplified in solid tumors (e.g., non-small cell lung cancer (NSCLC), breast cancer, melanoma) and hematologic malignancies, playing a key role in therapy resistance [26,27,28]. Co-overexpression of multiple anti-apoptotic proteins is common in cancers such as multiple myeloma, AML, triple-negative breast cancer (TNBC), melanoma, and glioblastoma, often requiring dual or multi-targeted strategies [29,30,31]. These insights led to the development of BH3 mimetics, a class of drugs that selectively inhibit anti-apoptotic BCL2 proteins. The first validated compound, ABT-737, emerged in 2005, and the field advanced with the FDA approval of venetoclax in 2016. Figure 2 and Table 2 list BH3 mimetics in clinical use or trials, while Table 3 details preclinical compounds.

### 2.3. Modulating BCL2 Family of Proteins with BH3 Mimetics for Cancer Treatments

BH3 mimetics are small molecules that mimic BH3-only pro-apoptotic proteins to selectively inhibit anti-apoptotic BCL2 family members. By binding to the hydrophobic groove of proteins like BCL2, BCLXL, and MCL1, they prevent sequestration of pro-apoptotic partners, enabling activation of BAX/BAK, MOMP, and apoptosis. This mechanism bypasses upstream resistance, such as p53 loss, making BH3 mimetics a promising strategy for cancers reliant on anti-apoptotic BCL2 proteins [32,33,34,35].

Drug development has been challenging due to the structural similarity among BCL2 family proteins, which complicates selective inhibition and increases toxicity risk. Nonetheless, decades of research have led to several BH3 mimetics reaching clinical use or trials. Most target either BCL2 or MCL1. Venetoclax (ABT-199), a BCL2 inhibitor, has shown strong efficacy in CLL and AML [36,37,38,39,40,41,42]. MCL1 inhibitors like AMG 176, AZD5991, and S64315 are in trials for multiple myeloma, AML, and solid tumors [32,43,44,45]. Navitoclax (ABT-263) targets BCL2, BCLXL, and BCLW and is being tested in solid tumors and T cell lymphomas [46,47,48] and solid cancers [49].

BCL2A1 inhibitors are under development but not yet in clinical trials. BCL2A1 is tightly regulated in normal tissues but often overexpressed in cancers such as AML, CLL, DLBCL, breast cancer, melanoma, and lung cancer. Its shallow binding pocket poses a challenge for drug design [50,51,52].

### 2.4. Clinical Outcomes of BH3 Mimetics, Adverse Effects, and Future Directions

Venetoclax is the most successful BH3 mimetic to date, showing strong efficacy in hematologic malignancies, particularly CLL and AML. In CLL, it is FDA-approved as a fixed-duration therapy in combination with obinutuzumab or rituximab. In relapsed or refractory CLL, venetoclax plus rituximab achieved an overall response rate of 84%, including 41% complete responses [39]. The combination of venetoclax–obinutuzumab is also FDA-approved for treatment-naïve CLL patients with comorbidities.

In AML, venetoclax is used with hypomethylating agents (azacitidine or decitabine) or low-dose cytarabine, significantly improving outcomes compared to monotherapy [53,54]. These regimens have become key treatment options for older or unfit AML patients.

Common side effects of venetoclax include tumor lysis syndrome, neutropenia, infections, gastrointestinal symptoms (nausea, diarrhea, constipation), and autoimmune hemolytic anemia [55]. While the safety profile is generally manageable in CLL patients, AML patients require closer monitoring for cytopenia [56]. However, BCL2 inhibitors alone, like venetoclax, have limited efficacy in solid tumors, which often depend on MCL1 and BCLXL for survival [31,57,58,59].

The immunomodulatory properties of BH3 mimetics and their potential synergy with immunotherapies or immunomodulatory agents represent a promising avenue for future cancer treatment (see details in Section 4.3 below). Several clinical trials are currently exploring such combinations, especially with venetoclax. For example, venetoclax is being tested with the immunomodulatory agents lenalidomide and rituximab in lymphomas (NCT04447716). A Phase Ib trial is evaluating pembrolizumab with decitabine, with or without venetoclax, in patients with AML or myelodysplastic syndrome (MDS) (NCT03969446). Another study is investigating a PD-1 inhibitor-based triple regimen (venetoclax + hypomethylating agent + PD-1 inhibitor) in relapsed/refractory AML and high-risk MDS (NCT06536959). Venetoclax has also been combined with pembrolizumab in NSCLC (NCT04274907), although this study was terminated for strategic reasons.

ABT-263 (Navitoclax) has shown promising efficacy in some clinical trials for hematologic malignancies [60]. However, its clinical development has been constrained by dose-limiting toxicities, particularly thrombocytopenia resulting from BCLXL inhibition in platelets [46,61]. Despite these challenges, several clinical trials—both completed and ongoing—have explored navitoclax in hematologic and solid tumors. Current trials include combinations with BRAF or MEK in melanoma (NCT01989585). One of the recently concluded clinical trials of navitoclax in combination with trametinib in RAS-mutant gynecologic cancer indicates the combination to be tolerable, and durable clinical responses were observed in patients (NCT02079740) [62]. Future directions include developing more selective BCL2 family inhibitors, mitigating thrombocytopenia, and investigating navitoclax in solid tumors and aging-related diseases, where senolytic activity may offer therapeutic benefit.

For MCL1 inhibitors, while preclinical activity is encouraging, clinical use has been hindered by on-target cardiac toxicity [63]. Some trials have been completed, although several were terminated due to safety concerns. S64315 (MIK-665) is the most successful MCL1 inhibitor to date. For instance, a Phase I study of intravenously administered S64315 in combination with venetoclax in AML patients has been completed (NCT03672695). In another trial, the MCL1 inhibitor PRT1419 demonstrated acceptable safety and tolerability in patients with advanced or metastatic solid tumors, with common adverse events including neutropenia, nausea, diarrhea, and vomiting, and notably without cardiac toxicity (NCT04837677 [64]). However, subsequent studies of PRT1419 alone or in combination with azacitidine or venetoclax were terminated (NCT05107856).

The most frequently reported and concerning adverse effects for MCL1 inhibitors were cardiotoxicity [63]. This has led the FDA to place holds on several clinical trials, including those for AMG-397, AMG-176, and AZD5991. Notably, there are no publicly available reports detailing the exact nature of the cardiotoxicity observed. Preclinical data suggest that MCL1 inhibition affects human cardiomyocytes, potentially due to MCL1’s non-apoptotic role in regulating mitochondrial dynamics [63]. Specifically, MCL1 inhibitor treatment on human cardiomyocytes alters functional parameters such as spike amplitude, beat propagation, and conduction velocity, which overlap with disruption of the mitochondrial and actin networks, ultimately leading to cell death.

Currently, there are no established protocols for preventing these cardiac side effects. However, general approaches used for preventing chemotherapy-induced cardiotoxicity, such as close monitoring, supportive cardiac medications, and intermittent dosing [65]. Further research into cardioprotective strategies may be applicable to MCL1 inhibitors. Another promising approach involves the development of next-generation MCL1 inhibitors that are potent yet short-lived, allowing rapid clearance from the body and minimizing cardiac exposure. For example, Rauh et al. reported that the short-lived MCL1 inhibitor BRD-810 demonstrated strong anti-tumor activity in preclinical models without obvious adverse effects on cardiomyocytes [66].

Moving forward, careful mitigation strategies and rational clinical designs will be critical for advancing BH3 mimetics. Overcoming the challenges requires rational drug design, biomarker-guided therapy, and toxicity mitigation. Current strategies focus on advanced delivery systems to improve selectivity and reduce side effects. Next-generation BH3 mimetics, including PROTACs, dual inhibitors, and nanoparticle formulations, aim to expand their therapeutic potential across diverse cancers [29,30,31,67,68].

## 3. Resistance Mechanisms of BH3 Mimetics

Resistance to BH3 mimetics occurs through various mechanisms. This review specifically focuses on resistance to BCL2 and MCL1 inhibitors, given their clinical relevance in cancer therapy.

### 3.1. Resistance to Venetoclax

**Figure 3:**
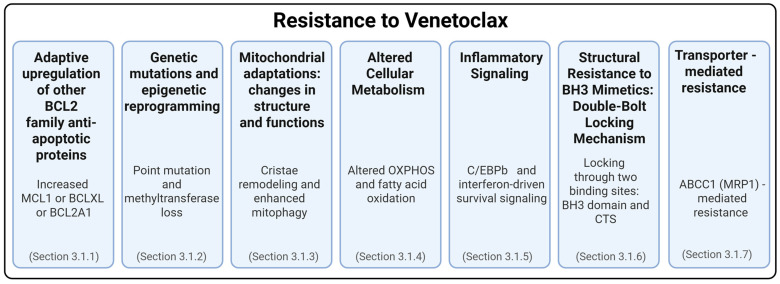
Multiple mechanisms of resistance to venetoclax.

Venetoclax, the only FDA-approved BH3 mimetic, has demonstrated strong clinical efficacy in treating hematologic malignancies by targeting the anti-apoptotic protein BCL2. Despite its initial success, long-term effectiveness is often compromised by the emergence of resistance [69]. This section explores the diverse mechanisms by which cancer cells evade venetoclax-induced apoptosis, including the upregulation of alternative anti-apoptotic proteins, mitochondrial and metabolic reprogramming, genetic and epigenetic alterations, and inflammatory signaling pathways [70]. These adaptations, summarized in Figure 3, highlight the complexity of resistance and the need for combination strategies to overcome therapeutic limitations.

#### 3.1.1. Adaptive Upregulation of Other BCL2 Family Anti-Apoptotic Proteins

A well-characterized resistance mechanism involves the compensatory upregulation of other anti-apoptotic BCL2 family members. In response to venetoclax-induced pressure, cancer cells often shift their apoptotic dependencies by increasing expression of MCL1, BCLXL, or BCL2A1 [71]. This adaptive response is frequently driven by oncogenic mutations in signaling molecules such as *KRAS*, *FLT3-ITD*, and *PTPN11*, which activate pro-survival pathways [72,73,74,75].

Disruption of the p53 pathway through MDM2 or XPO1 further contributes to resistance by elevating c-Myc, a key transcriptional driver of MCL1 and BCLXL [76]. In monocytic AML, high expression of C/EBPβ also promotes transcription of BCLXL and BCL2A1, reinforcing this escape mechanism [71]. These findings underscore how upstream oncogenic and transcriptional alterations can rewire apoptotic dependencies in AML.

#### 3.1.2. Genetic Mutations and Epigenetic Reprogramming in Venetoclax Resistance

Recent studies have uncovered a growing number of genetic and epigenetic mechanisms that contribute to resistance against venetoclax. Point mutations in *BCL2*—notably Asp103Glu, Phe104Leu, and Val148Leu—have been shown to significantly reduce venetoclax binding affinity, thereby impairing its ability to induce apoptosis [77,78]. Similarly, a therapy-induced mutation in *BAX* (P168A) disrupts the α9 helix, impairing mitochondrial targeting and preventing MOMP, a critical step in apoptosis initiation [79].

Beyond point mutations, epigenetic reprogramming also plays a pivotal role in venetoclax resistance [80]. Loss of the histone methyltransferase SETD1B downregulates pro-apoptotic genes such as BIM and BIK, enabling BCL2-independent survival in lymphoma and AML models [81]. Complementing this, genome-wide CRISPR screens have identified ZNF740 as a novel resistance regulator; its deletion reduces NOXA expression, stabilizing MCL1 and attenuating apoptotic signaling [82].

Additionally, upregulation of RNA methylation enzymes NSUN1 and NSUN2 has been shown to alter RNA polymerase II activity, reshaping the transcriptional landscape to favor cell survival under therapeutic stress [83]. Together, these findings highlight the multifaceted nature of venetoclax resistance and underscore the importance of targeting genetic, transcriptional, and epigenetic vulnerabilities to enhance therapeutic efficacy.

#### 3.1.3. Mitochondrial Adaptations: Changes in Structure and Functions

Mitochondrial structural remodeling has emerged as a critical mechanism underlying resistance to venetoclax. Overexpression of the chaperone CLPB promotes cristae remodeling and maintains structural integrity under stress, limiting apoptotic signaling and enhancing cell survival in the presence of venetoclax [84,85]. Structural changes in resistant AML cells include denser cristae with narrowed lumens and elevated levels of CLPB and OPA1, which help maintain mitochondrial architecture and reduce apoptotic susceptibility [84,85,86].

Functional adaptations also contribute to resistance. Glytsou et al. [87] showed that mitophagy, selective mitochondrial degradation, contributes to resistance. This process is driven by upregulation of mitofusin-2, which facilitates mitochondrial fusion and acts as a receptor for Parkin-dependent mitophagy, involving the E3 ubiquitin ligase Parkin and the autophagy machinery [87,88]. Enhanced mitophagy correlates with the loss of pro-apoptotic mediators such as PUMA, TP53, BAX, and BAK, which are essential for MOMP and apoptosis initiation [89]. Upregulation of mitochondrial fitness regulators like TOMM70A and MARCH5 further supports survival, while their loss sensitizes AML cells to venetoclax [87,90,91].

Importantly, combining BH3 mimetics with autophagy inhibitors (e.g., chloroquine or ULK1 inhibitors) restores apoptotic sensitivity in preclinical models [87]. Targeting CLPB has also been shown to enhance venetoclax and venetoclax/azacitidine efficacy, even in p53-deficient contexts [85].

#### 3.1.4. Altered Cellular Metabolism

Metabolic plasticity represents another key mechanism of resistance. Venetoclax, particularly when combined with azacitidine, suppresses oxidative phosphorylation (OXPHOS) by inhibiting amino acid metabolism in leukemia stem cells [92]. However, resistant cells adapt by shifting their metabolic fuel source toward fatty acid oxidation [93]. Elevated levels of L-carnitine and acyl-carnitines mark this metabolic reprogramming, enabling sustained OXPHOS and therapeutic evasion [93].

#### 3.1.5. Inflammatory Signaling

Recently, inflammatory signaling networks have been recognized as critical resistance drivers. Allen and Bottomly et al. [71] described a self-reinforcing inflammatory loop in monocytic AML, in which venetoclax treatment induces C/EBPβ expression. C/EBPβ, in turn, promotes IL1β and TNF-α signaling, further amplifying its own expression and upregulating anti-apoptotic proteins such as MCL1 and BCLXL [71]. In parallel, elevated interferon-γ signaling has been observed in resistant monocytic and del(7q) AML subtypes. This signaling enhances the expression of pro-survival genes, including HLA Class I and II and interferon regulatory factors, while dampening apoptotic priming [94].

#### 3.1.6. Structural Resistance to BH3 Mimetics: The Double-Bolt Locking Mechanism

**Figure 4:**
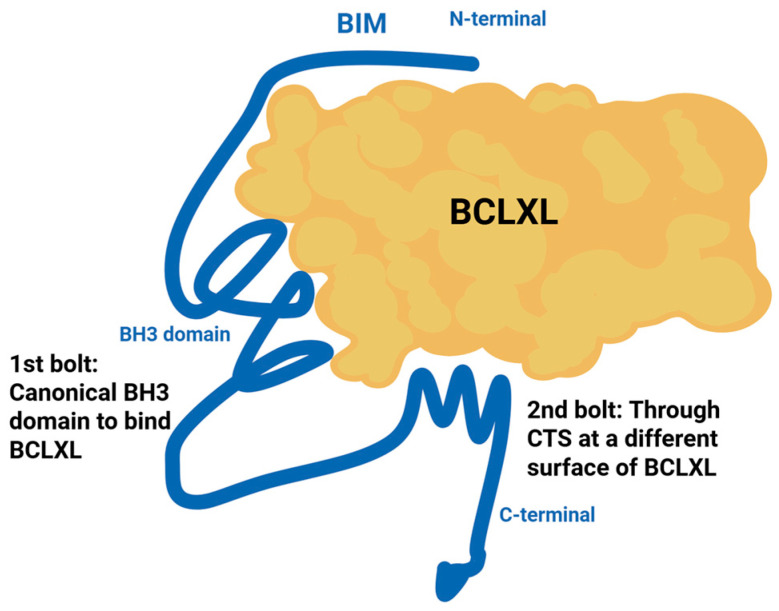
Schematic model of double-bolt locking mechanism. This diagram illustrates this structural resistance mechanism, using the interactions between BIM and BCLXL as an example. The first binding interface (“1st bolt”) involves the canonical BH3 domain of BIM engaging the hydrophobic groove of BCLXL—a site commonly targeted by BH3-mimetic drugs. The second interface (“2nd bolt”) is mediated by the C-terminal sequence (CTS) of BIM, which binds a distinct surface on BCLXL not targeted by BH3 mimetics. This dual engagement stabilizes BIM in a resistant complex, preventing its displacement by BH3 mimetics and thereby maintaining inhibition of apoptosis. Figure adapted from [95].

Recent studies have identified a structural resistance mechanism termed “double-bolt locking”, which enables BH3-only proteins such as BIM and PUMA to resist displacement by BH3 mimetics [95]. Figure 4 illustrates this mechanism using BIM and BCLXL as an example. These proteins interact with anti-apoptotic BCL2 family members (e.g., BCL2, BCLXL) through two distinct binding interfaces: the canonical BH3 domain, which inserts into the hydrophobic groove of the anti-apoptotic protein (“1st bolt”), and a second interaction mediated by their C-terminal sequences (“2nd bolt”). This dual engagement forms a highly stable complex that resists disruption by BH3 mimetics, which typically target only the BH3-binding groove.

Importantly, this resistance is not driven by mutation, but rather by inherent structural features of BIM and PUMA. The double-bolt locking mechanism stabilizes the BIM–BCL2/XL complex, preventing displacement by agents such as venetoclax or navitoclax, thereby neutralizing the pro-apoptotic activity of these mimetics [95].

#### 3.1.7. Transporter-Mediated Resistance to BH3 Mimetics

ABCC1 (MRP1), an ATP-binding cassette transporter, mediates resistance to BCL2 inhibitors in AML by actively exporting BH3 mimetics such as venetoclax, AZD4320, and navitoclax, thereby reducing their intracellular concentrations and efficacy [96]. CRISPR/Cas9 screening and pharmacologic inhibition studies confirmed that ABCC1 loss sensitizes AML cells to BCL2 inhibition, including in venetoclax-resistant models. High ABCC1 expression correlates with poor clinical response, supporting its role as a predictive biomarker and therapeutic target to overcome resistance.

### 3.2. Resistance to MCL1 Inhibitors

**Figure 5:**
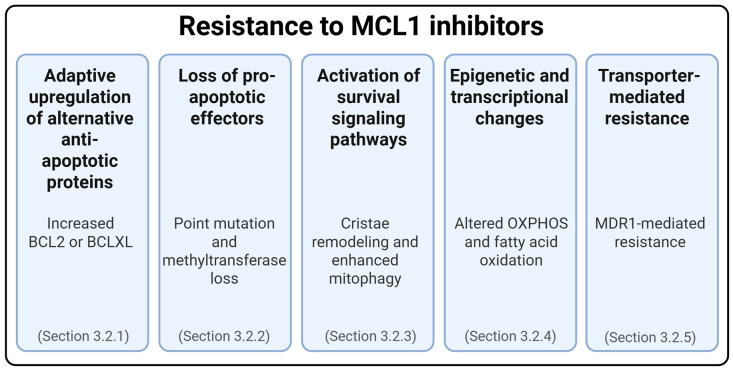
Multiple resistance mechanisms to MCL1 inhibitors.

Although resistance to MCL1 inhibitors is less well characterized than that to venetoclax, emerging evidence reveals both overlapping and distinct mechanisms. While resistance to both agents can involve upregulation of alternative anti-apoptotic proteins, MCL1 inhibitor resistance also includes unique adaptations such as the influence of the pro-apoptotic effector BAK, which binds more strongly to MCL1 than to BCL2 [97,98]. These changes allow cancer cells to bypass MCL1 dependency and evade apoptosis. Key mechanisms are illustrated in Figure 5 and detailed in the following subsections.

#### 3.2.1. Adaptive Upregulation of Alternative Anti-Apoptotic Proteins

Cancer cells can evade MCL1 inhibition by upregulating untargeted pro-survival BCL2 family members, particularly BCLXL and BCL2A1, enabling continued survival despite MCL1 blockade [99]. This compensatory shift has been documented in AML, multiple myeloma, NSCLC, and TNBC. For example, AML and myeloma models treated with the MCL1 inhibitor S63845 show increased expression of BCL2 and BCLXL, indicating a shift in apoptotic dependency [29,59]. Similarly, NSCLC and TNBC cell lines have shown transcriptional upregulation of BCLXL following MCL1 inhibition, blunting apoptotic responses [100,101,102]. These adaptive responses are driven by mechanisms such as mitochondrial priming, ER stress signaling, and transcriptional activation via STAT3 and AKT pathways. Combination strategies co-targeting MCL1 with BCL2 (e.g., venetoclax) or BCLXL (e.g., A-1331852) have shown synergistic apoptotic effects, supporting the rationale for dual inhibition in preclinical models [58,59,103,104,105,106,107].

Additionally, MCL1 inhibitor treatment may paradoxically induce MCL1 upregulation in AML, maintaining anti-apoptotic signaling and cell viability despite therapeutic pressure [108].

#### 3.2.2. Loss of Pro-Apoptotic Effectors

Another mechanism for the resistance to MCL1 inhibitor involves the loss of pro-apoptotic effectors such as BAX, BAK, or BIM. These proteins directly interact with MCL1 to initiate apoptosis, and their absence reduces apoptotic priming. BAK-deficient cells exhibit marked resistance to MCL1 inhibitors [109,110], while loss of BIM contributes to MCL1 resistance in lymphomas and myelomas [111,112]. Notably, deletion of BAX alone is sufficient to confer resistance to the MCL1 inhibitor S63845 in aggressive lymphoma models, even when BAK is intact, highlighting that BAX plays a non-redundant role in MCL1-dependent cell death [99].

NOXA is known to bind MCL1 but not BCL2, and promotes MCL1 degradation [113]. Targeted therapy-induced NOXA downregulation lowers MCL1 priming and drives adaptive resistance through enhanced MCL1 function [31,114,115]. However, its role in resistance to MCL1 inhibitors has not been systematically investigated.

#### 3.2.3. Activation of Survival Signaling Pathways

Resistance to MCL1 inhibitors can also be mediated by pro-survival signaling pathways, such as the PI3K/AKT, ERK, or JAK/STAT. These pathways promote survival through transcriptional or translational upregulation of BCL2 family proteins, or by preventing apoptosis through non-mitochondrial mechanisms [115,116,117]. In AML, elevated c-myc and MCL1 contribute to resistance, and targeting c-Myc by 10058-F has been proposed as a feasible approach to overcome MCL1 inhibitor resistance [118]. Activation of these pathways has been linked to reduced sensitivity to MCL1 inhibition in both solid tumors and hematologic malignancies.

#### 3.2.4. Epigenetic and Transcriptional Changes

Loss of SETD1B emerges as a shared resistance mechanism across BH3 mimetics, reducing expression of pro-apoptotic genes regardless of the targeted anti-apoptotic protein [81]. This points to a core epigenetic vulnerability that can drive cross-resistance between BH3 mimetics. Mutations in AML, such as WT1 and BCORL1, can confer innate or acquired resistance to MCL1 inhibitors, reducing treatment efficacy [119]. Further upstream, *KRAS* and *PTPN11* mutations contribute to resistance by activating signaling cascades that increase MCL1 expression transcriptionally [73,120].

#### 3.2.5. Transporter-Mediated Cross-Resistance to MCL1 Inhibitors

MDR1 (also known as P-glycoprotein or ABCB1) can contribute to resistance against MCL1 inhibitors by actively exporting these agents from cancer cells, reducing their intracellular levels and therapeutic efficacy [121]. Cells with elevated MDR1 expression can survive lethal doses of MCL1 inhibitors due to enhanced drug efflux. This mechanism adds a layer of protection against apoptosis and underscores the importance of monitoring MDR1 status in tumors treated with MCL1 inhibitors. Co-administration of MDR1 inhibitors or development of compounds that evade MDR1-mediated export may help overcome resistance, particularly in MDR1-high multiple myeloma.

### 3.3. Shared and Distinct Mechanisms of Resistance to Venetoclax and MCL1 Inhibitors

As discussed above, venetoclax and MCL1 inhibitors each have distinct resistance mechanisms. Here, we compare and contrast their shared and unique features to highlight broader patterns across BH3 mimetics.

Resistance to venetoclax and MCL1 inhibitors involves several common mechanisms. A central mechanism involves compensatory upregulation of alternative anti-apoptotic proteins: inhibition of BCL2 often leads to increased MCL1, BCL2A1, or BCLXL expression [71], while MCL1 inhibition can upregulate BCL2 or BCLXL [99,100,101,102]. Both drug classes also rely on intact pro-apoptotic effectors, so loss or dysfunction of BAX, BAK, or BH3-only proteins such as BIM and PUMA can drive cross-resistance.

Epigenetic alterations, such as SETD1B loss, reduce apoptotic priming and contribute to resistance in both contexts [81]. Transporter-mediated efflux is another shared mechanism, with ABCC1 implicated in venetoclax resistance [96] and MDR1 in resistance to MCL1 inhibitors [121]. 

The “double-bolt” mechanism described for BIM-BCL2/XL interactions [95] likely extends to BIM-MCL1 complexes as well; based on that, BIM also interacts with MCL1 [122], suggesting a structural basis for MCL1 inhibitor resistance, too.

In addition to these common pathways, each BH3 mimetic also faces unique resistance mechanisms. Venetoclax resistance is frequently associated with BCL2 mutations (e.g., Gly101Val, Asp103Glu) [77,78] and inflammatory signaling loops that drive MCL1 expression [71]. In contrast, resistance to MCL1 inhibitors is more often linked to STAT3-driven transcriptional programs [58,59,103,104,105,106,107] and selective loss of BAK [109,110].

Together, these overlapping and unique adaptations underscore the need for rational combination strategies to overcome resistance to BH3 mimetics.

### 3.4. Resistance Mechanisms to BH3 Mimetics in Solid Tumors

While resistance to BH3 mimetics has been extensively studied in hematologic malignancies, many of the underlying mechanisms—such as compensatory upregulation of alternative anti-apoptotic proteins, mutations in target proteins, and influences from the tumor microenvironment—are increasingly recognized as relevant in solid tumors as well.

Solid tumors, however, also exhibit intrinsic resistance to BH3 mimetics due to distinct apoptotic dependencies. For example, BCLXL and MCL1 are critical for cell survival in melanoma [31,57], rendering them inherently resistant to BCL2 inhibitors alone [58,59].

Importantly, cell-type-specific transcriptional programs also shape apoptotic dependencies and drug responses. For instance, the melanocyte lineage transcription factor MITF can regulate the expression of BCL2 [123] and BCL2A1 [124], creating a robust survival network that limits the efficacy of single-agent BH3 mimetics. Interestingly, uveal melanoma displays a distinct apoptotic profile, characterized by low BCL2A1 and high PUMA expression, which correlates with increased sensitivity to MCL1 inhibitors [105]. These differences underscore the importance of molecular context and tumor subtype in determining BH3 mimetic responsiveness.

Together, these findings suggest that overcoming resistance in solid tumors will require combinatorial targeting of multiple anti-apoptotic BCL2 family members, guided by functional profiling and transcriptional context.

In summary, the findings in Section 3 underscore the multifaceted and evolving nature of resistance to BH3 mimetics, and highlight emerging therapeutic targets—genetic, epigenetic, mitochondrial, metabolic, and inflammatory—for overcoming treatment failure.

## 4. Combination Approaches to Improve Efficacy of Treating Cancers

**Figure 6:**
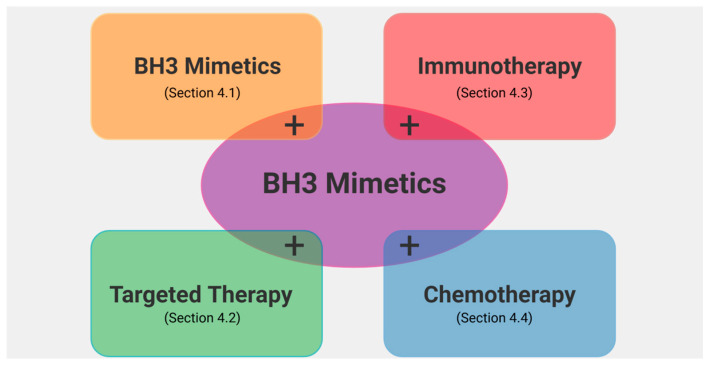
Combination strategies of BH3 mimetics in treating cancers. “+” indicates “in combination with”. For example, BH3 mimetics in combination with targeted therapy.

BH3 mimetics have revolutionized cancer therapy by targeting anti-apoptotic BCL2 family proteins to induce cell death. However, the efficacy of single-drug treatment is often limited by resistance mechanisms (see Section 3). Combining BH3 mimetics with other therapies, such as other BH3 mimetics, immune checkpoint inhibitors, targeted drugs, or chemotherapy, can help overcome resistance by enhancing apoptosis, disrupting survival pathways, and improving immune responses (Figure 6). These combination strategies broaden the therapeutic potential of BH3 mimetics across diverse cancer types.

### 4.1. BH3 Mimetics in Combination with Other BH3 Mimetics

Combining BH3 mimetics that target multiple BCL2 family proteins is an effective strategy to overcome resistance in both hematologic and solid tumors [58,125]. Tumors often evade apoptosis through adaptive upregulation of alternative anti-apoptotic proteins when one is inhibited [71]. Dual targeting, such as venetoclax with MCL1 inhibitors, has shown synergistic effects in AML and MDS, including venetoclax-resistant cells [126,127]. Inhibiting BCL2A1 can also restore venetoclax sensitivity, highlighting the value of co-targeting resistance mechanisms [128].

Dual-targeting strategies are also effective in solid tumors like melanoma, which often express multiple anti-apoptotic proteins. Co-inhibition of MCL1 with BCL2 or BCLXL induces apoptosis in both bulk melanoma cells and therapy-resistant melanoma-initiating cells [58,59]. A triple combination of S63845, venetoclax, and parthenolide (a NOXA inducer and MCL1 suppressor) further enhances apoptosis by suppressing MITF, a key regulator of melanoma plasticity. While MITF-high cells are proliferative, MITF-low cells are invasive and resistant, so co-targeting these populations led to deeper responses [129,130].

In summary, combinations of multiple BH3 mimetics offer a promising strategy to overcome apoptotic resistance. Their clinical success will depend on rational design, biomarker-guided patient selection, and effective toxicity management.

### 4.2. BH3 Mimetics with Targeted Therapy

Integrating BH3 mimetics with targeted therapies enhances apoptosis in cancers driven by specific oncogenic pathways. In FLT3-ITD AML, combining venetoclax with FLT3/MERTK inhibitors (e.g., MRX-2843) suppresses MCL1, disrupts metabolism, and spares normal hematopoietic cells [131]. Similarly, venetoclax + sitravatinib reduces MAPK/ERK signaling and downregulates MCL1/BCLXL, improving leukemia clearance and survival [132]. In CLL, APG-115 (an MDM2 inhibitor) restored p53 activity, enhancing venetoclax efficacy in TP53 wildtypes [133].

BH3 mimetics also synergize with epigenetic therapy drugs, another form of targeted therapy. In multiple myeloma and melanoma, MCL1 inhibitors like S63845 combined with HDAC inhibitors or redox modulators upregulate pro-apoptotic proteins like BIM and NOXA, resensitizing resistant cells [130,134]. In another study, JQ1 (BET inhibitor) sensitized rhabdomyosarcoma cells to BCLXL and MCL1 inhibitors by rewiring apoptotic gene expression [135].

Targeting survival pathways further enhances BH3 mimetic efficacy. PI3Kδ inhibition with roginolisib boosts venetoclax activity by degrading MCL1 and inducing FOXO1-driven BIM expression, with clinical trials underway in CLL [136] (NCT04328844). In ALK^+^ anaplastic large-cell lymphoma, combining crizotinib with BCL2/BCLXL inhibition overcame treatment resistance [137]. In aggressive DLBCL, co-targeting PIM kinases with venetoclax showed strong synergy [138]. In APC-mutant colorectal cancer, GSK-3 inhibition enhanced WNT signaling, sensitizing tumors to BCLXL blockade and selectively eliminating mutant organoids [139].

Metabolic vulnerabilities also offer opportunities to enhance BH3 mimetic efficacy. In AML, SIRT3-driven fatty acid oxidation supports venetoclax resistance in leukemic stem cells; inhibiting SIRT3 with YC8-02 induces lipotoxicity and sensitizes resistant cells while sparing healthy progenitors [140]. Mitochondrial inhibitors like ME-344 disrupt OXPHOS and reduce MCL1 expression, further enhancing venetoclax activity [141]. In DLBCL, glutaminase-1 inhibition amplifies venetoclax-induced apoptosis by increasing metabolic stress [142]. These findings highlight how targeting genetic, epigenetic, signaling, and metabolic pathways can synergize with BH3 mimetics to overcome resistance, restore apoptosis, and expand their clinical potential.

### 4.3. BH3 Mimetics Enhance Immunotherapies

Combining BH3 mimetics with immune checkpoint inhibitors (ICIs) or immunomodulatory agents represents a promising new approach to overcoming resistance and boosting the effectiveness of cancer immunotherapy. These combinations improve immune cell function and increase tumor cell susceptibility to immune-mediated attack. This section outlines how BH3 mimetics reshape the immune microenvironment and potentiate immune cell-mediated tumor elimination, with examples spanning checkpoint blockade, immune-modulating drugs, and adoptive cell therapies.

BH3 mimetics, such as venetoclax and MCL1 inhibitors, can reprogram the tumor immune microenvironment to enhance ICI responses. These effects include promoting T cell activation [143], reprogramming macrophages [144], and reducing immunosuppressive subsets [145]. These changes collectively enhance the efficacy of ICIs such as anti-PD-1 treatment. For example:

- Venetoclax increased CD8^+^ T cell infiltration and effector function without affecting T cell viability, leading to improved responses to anti-PD-1 in preclinical models [143].
- The BCL2 inhibitor APG-2575 reprogrammed macrophages from an M2-like (immunosuppressive) to M1-like (pro-inflammatory) phenotype via NLRP3 activation, significantly enhancing CD8^+^ T cell infiltration and the anti-tumor activity of PD-1 blockade in solid tumor models [144].
- In lymphoma, venetoclax combined with anti-CD47 antibodies (e.g., TJC4) enhanced macrophage-mediated phagocytosis and demonstrated synergistic immune activation [146].
- In AML, venetoclax plus hypomethylating agents (HMAs) increases PD-1 expression on T cells—a reversible effect with nivolumab co-treatment—resulting in restored T cell function and reduced disease burden [147].
- MCL1 inhibitors such as S64315 have also shown immune-modulating effects by depleting myeloid-derived suppressor cells, thereby alleviating immunosuppression and enhancing ICI efficacy in melanoma models [145].

Together, these combination strategies reprogram the immune landscape and amplify T cell-mediated tumor killing, often without adding significant toxicity [146,148].

In addition to modulating the immune cells, BH3 mimetics sensitize tumor cells to immune-mediated apoptosis by lowering their apoptotic threshold—especially under inflammatory conditions or during ICI treatments. ICIs elevate interferon-γ (IFN-γ) signaling, which upregulates BH3-only proteins in tumor cells, priming them for apoptosis. BH3 mimetics such as venetoclax exploit this state to trigger mitophagy and apoptosis without harming immune effector cells [148].

Additional synergistic strategies include the following:

- BH3 mimetics with STING agonists: Particularly effective in TP53-mutant tumors, this combination restores apoptotic signaling via innate immune pathways, even in the absence of functional p53, and enhances tumor killing with minimal toxicity [149].
- BH3 mimetics with antiretrovirals: In HTLV-1–driven leukemia, MCL1 inhibition selectively eliminated infected T cells and slowed disease progression, suggesting a potential curative approach [150].
- Adoptive cell therapies: In CAR T and NK cell therapies, venetoclax sensitizes tumor cells to killing while sparing immune cells [151,152,153]. Engineering CAR T cells with BCL2 mutations protects them from venetoclax-induced apoptosis, enabling safe combination strategies even in resistant settings.

**Figure 7:**
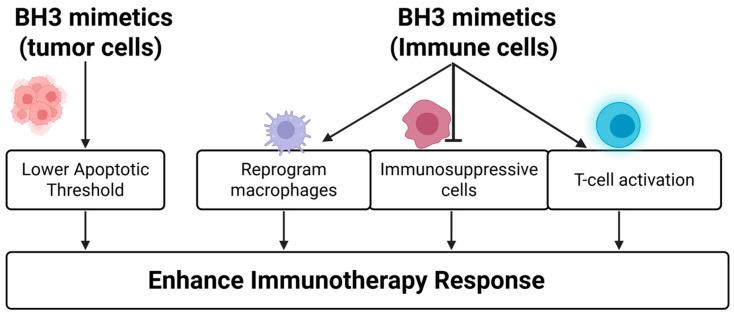
A novel strategy to enhance the efficacy of cancer immunotherapies by combining them with BH3 mimetics. BH3 mimetics exert two effects: (1) they directly lower the apoptotic threshold of tumor cells, making them more responsive to immunotherapy, and (2) they modulate immune cells by reprogramming macrophages, inhibiting immunosuppressive cells such as MDSCs, and activating T cells. Together, these effects increase the efficacy of immunotherapies through direct and indirect ways to promote tumor cell killing.

Overall, BH3 mimetics enhance immunotherapy through dual mechanisms: modulating immune cell function and sensitizing tumor cells to apoptosis. Figure 7 illustrates how these agents act on both compartments to improve therapeutic responses. These synergistic effects provide a compelling rationale for continued clinical development aimed at overcoming resistance and improving outcomes across diverse cancer types.

### 4.4. BH3 Mimetics with Chemotherapy

Combining BH3 mimetics with conventional chemotherapy represents a strategic approach to overcoming chemoresistance by directly targeting cancer cell survival mechanisms. Many chemotherapeutics inadvertently induce both pro-apoptotic and pro-survival signals, creating a paradoxical state where apoptosis is initiated but blocked. BH3 mimetics help tip the balance toward cell death by neutralizing anti-apoptotic BCL2 family proteins. This therapeutic mechanism has been shown to enhance cytotoxicity in both hematologic and solid tumors.

In hematologic cancers like AML and ALL, chemotherapy often lowers MCL1 levels, increasing reliance on BCL2, thereby sensitizing cells to venetoclax. Preclinical and early clinical studies have shown venetoclax enhances the efficacy of chemotherapeutics such as asparaginase, cytarabine, and daunorubicin, improving remission rates without added toxicity [154,155,156].

In solid tumors such as TNBC, lung cancer, and mesothelioma, resistance to chemotherapy is frequently driven by MCL1 or BCLXL upregulation [157]. Combining BH3 mimetics like S63845 or navitoclax with cisplatin or paclitaxel has shown increased apoptosis and tumor suppression [158].

Overall, pairing BH3 mimetics with chemotherapy enhances efficacy across cancer types. Success depends on selecting the right BH3 mimetic based on tumor survival dependencies and managing toxicity to optimize outcomes.

## 5. Role of BH3 Mimetics Beyond Cancer

**Figure 8:**
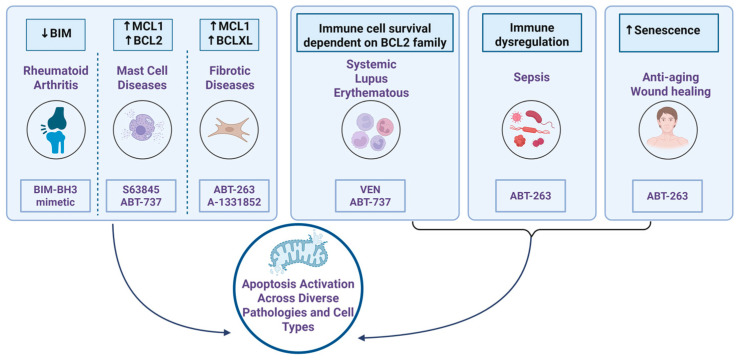
The role of BH3 mimetics in treating diseases other than cancers. BH3 mimetics have been explored for diseases such as rheumatoid arthritis, mast cell diseases, fibrotic disease, systemic lupus erythematosus, sepsis, and anti-aging. Upward and downward arrows within boxes indicate increased or deceased activity, respectively.

BH3 mimetics are best known for inducing apoptosis in cancer cells, but emerging evidence suggests they also modulate immune cell survival and function. This opens new therapeutic possibilities beyond oncology. By influencing immune responses, BH3 mimetics may offer benefits in conditions such as autoimmune diseases, fibrosis, and sepsis. This section highlights recent insights into their role as immune modulators in non-cancer settings (Figure 8), pointing to novel applications under active investigation.

### 5.1. Autoimmune Diseases

Dysregulated apoptosis, particularly involving the BCL2 protein family, contributes to the pathogenesis of autoimmune diseases such as rheumatoid arthritis (RA) and systemic lupus erythematosus (SLE) [159]. In RA, synovial tissues exhibit elevated expression of anti-apoptotic BCL2 family proteins, blocking the programmed death of infiltrating immune cells and sustaining chronic inflammation [45,160,161]. Concurrently, the pro-apoptotic protein BIM is downregulated in RA synovium compared to osteoarthritis controls, further shifting the balance toward cell survival [162]. BH3 mimetics that mimic BIM can restore apoptosis and reduce disease severity [163]. Interestingly, this dysregulation appears localized to inflamed tissue, as peripheral blood cells in RA patients show no significant difference in BCL2 family expression compared to healthy controls [164].

In SLE, overexpression of BCL2, BCLXL, and Beclin in lymphocytes contributes to defective apoptosis and prolonged survival of autoreactive cells, while reduced BIM and BAX expression further skews the balance toward cell survival [164,165]. Preclinical studies show that BH3 mimetics such as ABT-737, which targets BCL2, BCLXL, and BCLW, can suppress T and B cell proliferation in lupus and arthritis models [166]. Clinically, venetoclax has also shown promise in SLE by reducing total lymphocytes and pathogenic antigen-experienced B cells, while maintaining good tolerability [167] NCT01686555).

Together, these findings highlight the potential of BH3 mimetics as immune modulators in autoimmune diseases and support further investigation beyond oncology.

### 5.2. Fibrotic Disease

Fibrosis is characterized by the excessive accumulation of fibrous connective tissue (scar tissue) in organs or tissues, typically resulting from injury, inflammation, or chronic disease [168]. BH3 mimetics have recently emerged as promising antifibrotic agents by selectively inducing apoptosis in myofibroblasts, the key drivers of fibrosis [19,169]. For example, navitoclax has shown significant efficacy in reducing fibrosis in conditions such as scleroderma by promoting myofibroblast death [170]. Similarly, A-1331852 markedly reduced fibrosis-inducing growth factors secreted by senescent cholangiocytes in primary sclerosing cholangitis [171]. These findings underscore the potential of BH3 mimetics to target survival pathways in fibrotic cells and offer new therapeutic avenues for treating fibrotic diseases.

### 5.3. Mast Cell Associated Diseases

Mast cells are tissue-resident immune cells found throughout the skin, gastrointestinal tract, and respiratory system, where they play central roles in allergic responses and chronic inflammation. In pathological conditions such as mastocytosis, asthma, and hypereosinophilic syndromes, mast cells often overexpress anti-apoptotic BCL2 family proteins, allowing them to evade apoptosis and persist in inflamed tissues. A comprehensive review highlights BH3 mimetics as a promising strategy to overcome these survival mechanisms [172]. Preclinical studies have shown that ABT-737 effectively reduces mast cell viability in both human and mouse models [173,174]. More recently, Odinius et al. demonstrated that the MCL1 inhibitor S63845 significantly decreased bone marrow progenitor cells in patients with hypereosinophilia [175]. These findings suggest that selective targeting of BCL2 family proteins could provide a novel therapeutic approach in mast cell– and eosinophil-driven diseases.

### 5.4. Targeting Senescent Cells: Regenerative and Anti-Aging Applications of BH3 Mimetics

BH3 mimetics are gaining recognition beyond oncology for their ability to selectively eliminate senescent cells, a key driver of age-related tissue dysfunction and impaired regeneration. Cellular senescence contributes to chronic inflammation, reduced tissue repair, and diminished stem cell function, making it a compelling target for therapeutic intervention.

Topical application of ABT-263 (navitoclax) in aged mouse skin reduces senescence markers such as p16, p21, and SA-β-gal, accelerating wound healing and restoring tissue vitality [176]. Similarly, pretreatment of synovial mesenchymal stem cells from osteoarthritis patients with ABT-263 selectively clears senescent cells, enhancing their chondrogenic and adipogenic differentiation potential [177].

By targeting cellular senescence, BH3 mimetics offer a promising strategy to rejuvenate tissues and improve the efficacy of stem cell-based therapies. These regenerative and anti-aging effects complement their roles in cancer immunotherapy and resistance modulation, underscoring the broad therapeutic potential of this drug class.

### 5.5. Sepsis

In addition to their regenerative effects, BH3 mimetics may also enhance immune resilience in aged individuals, particularly in the context of acute inflammatory conditions such as sepsis, especially in aged hosts where immune dysfunction is pronounced. The BH3 mimetic ABT-263 (navitoclax), known for its senolytic and anti-apoptotic properties, has demonstrated the ability to promote recovery from sepsis in aged mice. One report showed that ABT-263 not only inhibited autophagy but also enhanced macrophage function by increasing the expression of TREM-2 receptors and inducing Beclin-1-dependent autophagy [178]. These effects led to improved macrophage activity and protection against sepsis in aged hosts.

Overall, these findings suggest that beyond their established role in inducing cancer cell death, BH3 mimetics may hold therapeutic potential in immune-mediated disorders, regenerative and anti-aging medicine. Continued research could pave the way for novel BH3 mimetic-based treatment strategies in non-cancer diseases.

## 6. Non-Apoptotic Roles of BCL2 Family Proteins

While BCL2 family proteins are best known for regulating mitochondrial apoptosis, growing evidence reveals their involvement in a wide range of non-apoptotic physiological processes. These include mitochondrial metabolism, autophagy, calcium signaling, cell cycle regulation, and immune function (see Table 1 in Section 2). A comprehensive review [179] summarized some of these roles; here, we build on that foundation by presenting new findings from 2022 to 2025, with a focus on tissue-specific mechanisms and responses to pharmacologic stress. These emerging functions have important implications for both the efficacy and toxicity of BH3 mimetics.

Recent studies have identified MCL1 as a key regulator of mitochondrial metabolism, especially in fatty acid oxidation (FAO). MCL1 supports long-chain FAO through direct interaction with the enzyme ACSL1, maintaining energy production in metabolically active tissues like the heart and liver [180]. Genetic deletion or pharmacologic inhibition of MCL1 disrupts FAO, leading to metabolic collapse and tissue-specific toxicities, including cardiotoxicity and hepatotoxicity [180].

An in vivo study further demonstrated that substituting MCL1 with BCLXL or BCL2 rescued early embryonic lethality in mice but failed to restore MCL1’s metabolic functions during later development [181]. While BCLXL partially preserved OXPHOS and mitochondrial architecture, neither protein restored FAO, resulting in hepatic steatosis, metabolic dysfunction, and postnatal [181]. These findings suggest that some toxicities of MCL1 inhibitors may stem from disrupting its metabolic, not solely anti-apoptotic, functions.

Beyond metabolism, MCL1 also contributes to genome stability and cell cycle progression. A recent study showed that MCL1 localizes to the nucleus and interacts with the MCM helicase complex, a key regulator of DNA replication [182]. Inhibition of MCL1 led to replication stress, DNA damage, and reduced proliferation, even in cells resistant to apoptosis—suggesting that MCL1-targeting BH3 mimetics may also limit tumor growth via non-apoptotic mechanisms [182].

MCL1 also regulates autophagy and mitophagy, particularly in cardiomyocytes [183]. It promotes selective mitochondrial clearance while suppressing bulk autophagy under stress. Loss of MCL1 disrupts mitophagy, contributing to mitochondrial dysfunction and organ damage. This dual role in maintaining organelle quality may underlie some of the tissue-specific toxicities observed with MCL1 inhibitors.

Similarly, BCLXL, another anti-apoptotic protein, influences calcium homeostasis through the endoplasmic reticulum (ER) [184]. In cells expressing ER-localized BCLXL, IP3 receptor-mediated calcium release was suppressed, enhancing resistance to ER stress and moderating the unfolded protein response [184]. Thus, BH3 mimetics that disrupt BCLXL at the ER may inadvertently impair calcium signaling and contribute to off-target toxicity.

Beyond cell death and stress responses, BCLXL also modulates immune aging [185]. In transgenic mice, T cell-specific overexpression of BCLXL preserved mitochondrial integrity, reduced systemic inflammation, and delayed frailty onset during aging [185]. These effects were linked to improved T cell metabolism and homeostasis. While BCLXL inhibition is desirable in cancer, chronic suppression could impair immune resilience and tissue repair, particularly in elderly patients.

The non-apoptotic roles of BCL2 family proteins have significant clinical implications for BH3 mimetic development. For instance, MCL1 supports FAO in cardiomyocytes, and its inhibition disrupts energy metabolism, contributing to cardiotoxicity [63,180]. Similarly, BCLXL regulates ER calcium homeostasis, and its inhibition may trigger ER stress and impair cellular signaling [184].

These findings underscore the need to explicitly consider non-apoptotic functions of BCL2 family proteins in the clinical development of BH3 mimetics. Integrating metabolic, genomic, and immune biomarkers into early phase trials may help anticipate these effects, guide patient selection, and optimize dosing strategies. A deeper understanding of these non-apoptotic roles will be critical for designing safer and more effective therapies that minimize unintended toxicity while maximizing therapeutic benefit.

Collectively, these studies underscore a broader paradigm in which BCL2 family proteins act as key regulators of cellular metabolism, stress responses, and homeostasis, beyond their classical role in apoptosis. These non-apoptotic functions are often cell- and tissue-specific and may intersect with apoptotic pathways to influence therapeutic outcomes. As BH3 mimetics progress in clinical use, understanding and leveraging these non-apoptotic roles will be helpful for developing effective and well-tolerated therapies.

## 7. Target Selectivity and Biomarker-Driven Precision in BH3 Mimetic Therapy

As BH3 mimetics advance in clinical development, a key challenge is tailoring these agents to the apoptotic dependencies of specific cell types and disease contexts. The therapeutic efficacy of BH3 mimetics hinges on two core principles:

(1) Target selectivity—which anti-apoptotic BCL2 family protein the drug inhibits.
(2) Apoptotic priming—whether the target cells are already poised to undergo apoptosis.

**Figure 9:**
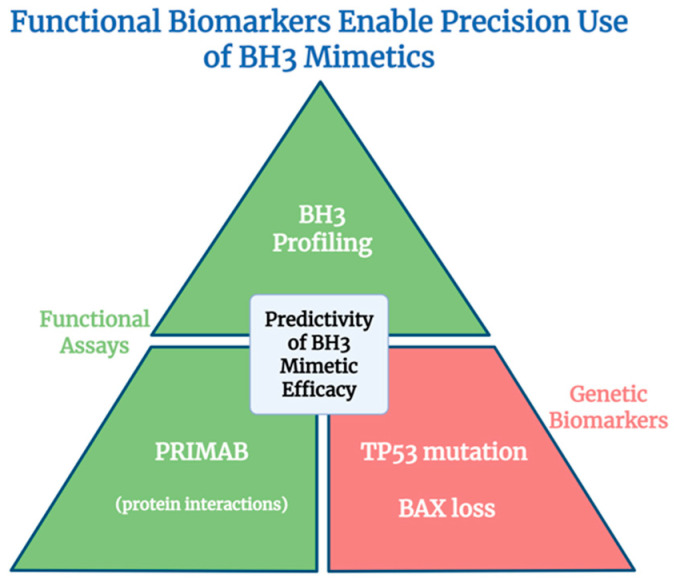
Predictive biomarkers guiding precision use of BH3 mimetics. Functional assays such as BH3 profiling and PRIMAB directly measure apoptotic priming and dependencies, while genetic biomarkers refine the prediction of drug efficacy. Together, these methods integrate drug selectivity with dynamic cellular and disease-specific contexts to optimize clinical use.

This section highlights how target selectivity and predictive biomarkers (Figure 9) are shaping precision strategies for BH3 mimetic therapies.

Different cancers exhibit distinct dependencies on BCL2 family proteins. For instance, venetoclax is highly effective in hematologic malignancies that rely on BCL2, whereas many solid tumors depend on MCL1 or BCLXL, requiring alternative inhibitors [186]. In neuroblastoma, BCLXL inhibition has demonstrated superior efficacy, with drug sensitivity more closely tied to functional interactions than protein expression levels [187].

Because these survival proteins are also expressed in healthy tissues, dose selection is critical to minimize toxicity. For instance, BCLXL inhibition can cause thrombocytopenia, and MCL1 inhibition may lead to cardiotoxicity. To mitigate these risks, short-acting agents like BRD-810, a transient MCL1 inhibitor, are being developed to reduce off-target effects [66]. Combination therapies targeting multiple BCL2 proteins show promise but require careful dosing to minimize overlapping toxicities [126,188,189,190].

Early biomarker strategies focused on measuring BCL2 family protein expression. However, expression levels alone do not reliably predict therapeutic response. NOXA, a natural MCL1 antagonist, has emerged as a promising marker: high NOXA levels predict sensitivity to BCLXL inhibitors, whereas low NOXA may require combination approaches [191].

Recent advances in functional and interaction-based technologies offer more precise tools for predicting drug response. BH3 profiling, a functional assay, assesses mitochondrial priming by exposing cells to synthetic BH3 peptides and measuring cytochrome c release, thereby revealing a cell’s dependency on specific survival proteins [188]. Despite its power, clinical implementation remains limited due to technical complexity.

A novel technology, PRIMAB, enables real-time measurement of endogenous protein–protein interactions using conformation-specific antibodies. It detects complexes such as BCL2-BIM, MCL1-BIM, and BCLXL-BIM in patient samples, providing a dynamic snapshot of apoptotic priming and drug sensitivity [192]. These tools support a precision medicine approach by aligning drug selection with evolving cellular vulnerabilities. However, its broader application is currently limited by the proprietary nature of the reagent generation process.

Genetic biomarkers also inform responses to BH3 mimetics. For example, TP53 mutations and BAX loss are associated with poor response to venetoclax in AML [76,99,149,193]. Circulating biomarkers—such as peripheral blood mononuclear cells, cell-free nucleic acids, and soluble proteins—offer a non-invasive approach to monitor dynamic changes in tumor biology and treatment response [194]. As our understanding of DNA-, RNA-, and protein-based indicators of apoptotic priming and anti-apoptotic dependencies improves, these analytes should be further explored as potential circulating biomarkers to guide and optimize BH3 mimetic therapy.

Together, integrating target selectivity with dynamic biomarkers provides a strong framework for the precision use of BH3 mimetics. As these therapies expand across disease settings, tailoring treatment strategies to individual cellular dependencies will be key to maximizing efficacy and minimizing toxicity.

## 8. Conclusions

BH3 mimetics have emerged as powerful tools to restore apoptosis in cancer and other diseases marked by pathological cell survival. Their clinical success in hematologic malignancies validates the therapeutic potential of targeting the BCL2 family. However, broader application is limited by resistance mechanisms, dose-limiting toxicities, and the complex, context-dependent biology of BCL2 family proteins.

Future progress hinges on biomarker-driven precision medicine, enabling tailored therapies based on apoptotic priming and survival dependencies. Combination strategies—with other BH3 mimetics, targeted agents, or immunotherapies—offer promising avenues to overcome resistance and enhance efficacy. Notably, BH3 mimetics can modulate the immune microenvironment, improving responses to checkpoint blockades and adoptive cell therapies.

Beyond oncology, BH3 mimetics show expanding promise in autoimmune, fibrotic, and infectious diseases, as well as in regenerative and anti-aging medicine. Their influence on non-apoptotic processes, including metabolism and immune aging, further broadens their therapeutic scope. Nonetheless, significant challenges persist: compensatory survival signaling, mitochondrial remodeling, and metabolic reprogramming continue to limit long-term efficacy, while toxicities such as thrombocytopenia and cardiotoxicity pose particular concerns.

Advancing the clinical impact of BH3 mimetics will require continued research integrating mechanistic insights, functional biomarkers, and rational combination strategies across diverse disease contexts.

## Figures and Tables

**Table 1:** List of BCL2 family, and their roles.

Name	Apoptotic Function	Non-Apoptotic Function
BCL2	Anti-apoptotic	Calcium homeostasis at the ER, metabolism, autophagy
MCL1	Anti-apoptotic	Calcium homeostasis at the ER, mitochondria shape, metabolism, and autophagy
BCLXL	Anti-apoptotic	Mitochondria shape, membrane permeability, calcium homeostasis at the ER, metabolism, autophagy, and DNA damage response
BCLW	Anti-apoptotic	Mitochondria shape
BCL2A1(BFL1)	Anti-apoptotic	Autophagy
BCLB	Primarily anti-apoptotic; may have cell-specific pro-apoptotic roles	Autophagy
BAD	Pro-apoptotic Initiator: Sensor	Autophagy, glucose, and lipid metabolism
BMF	Pro-apoptotic Initiator: Sensor	Autophagy
NOXA	Pro-apoptotic Initiator: Sensor	Glucose and lipid metabolism, autophagy
BID	Pro-apoptotic Initiator: Activator	Mitochondria shape, calcium homeostasis at the ER, unfolded protein response, DNA damage response, and glucose and lipid metabolism
BIM	Pro-apoptotic Initiator: Activator	Calcium homeostasis at the ER, unfolded protein response, and autophagy
PUMA	Pro-apoptotic Initiators: Activator	Calcium homeostasis at the ER, unfolded protein response, autophagy, and DNA damage response
BAX	Pro-apoptotic Executioner	Mitochondria shape, calcium homeostasis at the ER, metabolism, and unfolded protein response
BAK	Pro-apoptotic Executioner	Mitochondria shape, calcium homeostasis at the ER, metabolism, unfolded protein response, and autophagy
BOK	Pro-apoptotic Executioner	Calcium Homeostasis at the ER, unfolded protein response
BIK/BLK	Pro-apoptotic Executioner	Mitochondria shape, Calcium Homeostasis at the ER, Autophagy
HRK	Pro-apoptotic Initiators	Autophagy
BECLIN	Pro-autophagy	Autophagy
BNIP3	Pro-apoptotic Initiator: Sensor	Mitochondrial membrane permeability, calcium homeostasis, and autophagy

**Table 2:** BH3 mimetics that have entered clinical use or trials.

Compound Name	Description	Clinical Trials	Status as of 2025
**AT-101**	(-)-Gossypol; pan-BCL2 inhibitor derived from a natural product.	Phase I/II trials in prostate cancer, CLL, and others.	No recent trials: limited efficacy observed in past trials.
**Obatoclax (GX15-070)**	Pan-BCL2 family inhibitor; targets BCL2, BCXL, and MCL1; induces apoptosis.	Phase I/II trials in hematologic and solid tumors.	Development discontinued due to toxicity.
**Oblimersen sodium/G3139**	Antisense oligonucleotide targeting BCL2 mRNA to reduce BCL2 expression.	Multiple Phase III trials in CLL, melanoma, and others.	Clinical trials unsuccessful; development halted.
**ABT-263 (Navitoclax)**	Inhibits BCL2, BCLXL, and BCLW; induces apoptosis in cancer cells.	Phase I/II trials; major dose-limiting toxicity is thrombocytopenia.	Clinical trials ongoing.
**ABT199 (Venetoclax)**	Selective BCL2 inhibitor; FDA-approved for CLL, AML, and other cancers.	Multiple ongoing/completed Phase III trials.	FDA-approved for CLL, AML; Clinical trials ongoing for others.
**Pelcitoclax (APG1252)**	BCLXL/BCL2 dual inhibitor; aims to retain potency while reducing platelet toxicity.	Phase I/II trials in solid tumors and lymphomas in China and globally.	Clinical trial terminated.
**LP-118**	Potent BCL2-selective inhibitor designed to overcome venetoclax resistance.	Phase I trials in hematologic malignancies.	Clinical trials active and ongoing.
**TQB3909**	BCL2 inhibitor developed in China.	Phase I trials for hematologic cancers initiated in China.	Clinical trials ongoing but status unknown.
**S55746 (BCL201)**	Selective BCL2 inhibitor (developed by Servier/Novartis); orally bioavailable.	Phase I studies in hematologic malignancies; development status unclear.	Clinical trials completed.
**MIK665 (S64315)**	Selective MCL1 inhibitor (Servier/Novartis).	Phase I trials in AML, multiple myeloma, and solid tumors.	Majority of clinical trials completed.
**PRT1419**	Potent MCL1 inhibitor (Prelude Therapeutics).	Phase I clinical trials in hematologic malignancies.	Majority of clinical trials completed.
**Tapotoclax (AMG-176)**	Selective MCL1 inhibitor developed by Amgen.	Phase I trials in relapsed/refractory hematologic malignancies.	Clinical trial terminated.
**AMG-397**	Oral MCL1 inhibitor (Amgen); designed for convenience and improved delivery.	Phase I trials;	Clinical trial terminated.
**GS-9716**	MCL1 inhibitor developed by Gilead Sciences.	Phase I trials in hematologic malignancies;	Limited information: one listed trial but not recruiting.
**AZD5991**	Selective MCL1 inhibitor (AstraZeneca).	Phase I trials in hematologic cancers;	Clinical trial terminated due to cardiac toxicity concerns.
**ABBV-467**	MCL1 inhibitor developed by AbbVie.	Phase I trials	Clinical trial terminated.

**Table 3:** BH3 mimetics in the preclinical stage.

Compound Name	Description	Preclinical Studies
**HA141**	Early synthetic small molecule BCL2 inhibitor; binds the BH3-binding groove.	Demonstrated apoptosis induction in leukemia and glioma cell lines; limited by poor solubility and toxicity.
**ABT-737**	Potent inhibitor of BCL2, BCLXL, and BCLW; precursor to navitoclax.	Validated in multiple hematologic and solid tumor models; key tool in apoptosis research.
**WEHI-539**	Selective BCLXL inhibitor.	Used to dissect BCLXL-specific functions; effective in synergy with other agents in lymphoma models.
**A1155463/A1331852**	Selective, potent BCLXL inhibitors developed by AbbVie.	Induce apoptosis in BCLXL-dependent tumor cells; show synergy with chemotherapeutics.
**PROTAC BCLXL degrader (XZ424)**	PROTAC molecule designed to degrade BCLXL while sparing platelets by exploiting differential E3 ligase expression.	Effective in killing BCLXL-dependent cancer cells while minimizing thrombocytopenia in preclinical models.
**UMI-77**	MCL1-selective BH3 mimetic.	Shown to induce apoptosis in pancreatic and lung cancer models.
**S63845**	Selective and potent MCL1 inhibitor developed by Servier/Novartis.	Induces apoptosis in MCL1-dependent AML, multiple myeloma, and lymphoma models; widely studied preclinically.
**ANJ810**	MCL1 inhibitor developed by Anji Pharmaceuticals.	Preclinical data show antitumor activity in hematologic cancers; limited publicly available data.
**APG-3526/AS00491**	Selective BCL2 inhibitors developed by Ascentage Pharma.	Preclinical evidence suggests improved potency and resistance profile vs. venetoclax.
**STP-369**	MCL1 inhibitor.	Demonstrated tumor regression in preclinical lymphoma and AML models.
**Compound 56**	Covalent BCLXL inhibitor (structure-based design).	Reported to have improved selectivity and reduced platelet toxicity; still in the early preclinical phase.
**A-97 derivatives**	Series of small-molecule BCL2 inhibitors; A-97 is a potent BCL2 inhibitor.	Demonstrated pro-apoptotic activity in leukemia cells; derivatives under optimization.
**Silibinin**	Natural flavonoid with weak BH3-mimetic and anti-BCL2 activity.	Shown to induce apoptosis in various cancer cell lines; low potency; considered adjunctive or supportive.

## Data Availability

No new data were created or analyzed in this study. Data sharing is not applicable to this article.

## Abbreviations

The following abbreviations are used in this manuscript:

AML	Acute myeloid leukemia
BCL2	B-cell lymphoma 2
BCLXL	B-cell lymphoma 2 (BCL2) like-1 long
BCLW	Bcl-2-like protein 2
BCL2A1	Bcl-2-related protein A1
BH	Bcl-2 homology
BID	BH3-interacting domain death agonist
BIM	Bcl-2-interacting mediator of cell death
CLL	Chronic lymphocytic leukemia
DLBCL	Diffuse large B cell lymphoma
ER	Endoplasmic reticulum
FAO	Fatty acid oxidation
ICI	Immune checkpoint inhibitors
MCL1	Myeloid cell leukemia 1
MOMP	Outer mitochondrial membrane permeabilization
NOXA	Phorbol-12-myristate-13-acetate-induced protein 1
NSCLC	Non-small cell lung cancer
OXPHOS	Oxidative phosphorylation
PUMA	Bcl-2-binding component 3
TNBC	Triple-negative breast cancer
